# Addressing Stigma Through a Virtual Community for People Living with HIV: A Mixed Methods Study of the PositiveLinks Mobile Health Intervention

**DOI:** 10.1007/s10461-018-2174-6

**Published:** 2018-06-07

**Authors:** Tabor E. Flickinger, Claire DeBolt, Alice Xie, Alison Kosmacki, Marika Grabowski, Ava Lena Waldman, George Reynolds, Mark Conaway, Wendy F. Cohn, Karen Ingersoll, Rebecca Dillingham

**Affiliations:** 1 Department of Medicine, University of Virginia School of Medicine, Charlottesville, VA USA; 2 University of Virginia School of Medicine, Charlottesville, VA USA; 3 University of Virginia School of Nursing, Charlottesville, VA USA; 4 Health Decision Technologies, Oakland, CA USA; 5 Department of Public Health Sciences, University of Virginia School of Medicine, Charlottesville, VA USA; 6 Department of Psychiatry and Neurobehavioral Sciences, University of Virginia School of Medicine, Charlottesville, VA USA; 7 UVA Center for Global Health, P.O. Box 801379, Charlottesville, VA 22908 USA

**Keywords:** Stigma, Mobile health, Smartphone app, HIV/AIDS, PositiveLinks

## Abstract

Stigma has negative consequences for quality of life and HIV care outcomes. PositiveLinks is a mobile health intervention that includes a secure anonymous community message board (CMB). We investigated discussion of stigma and changes in stigma scores. Of 77 participants in our pilot, 63% were male, 49% Black, and 72% had incomes below the federal poverty level. Twenty-one percent of CMB posts (394/1834) contained stigma-related content including negative (experiencing stigma) and positive (overcoming stigma) posts addressing intrapersonal and interpersonal stigma. Higher baseline stigma was positively correlated with stress and negatively correlated with HIV care self-efficacy. 12-month data showed a trend toward more improved stigma scores for posters on the CMB versus non-posters (− 4.5 vs − 0.63) and for posters of stigma-related content versus other content (− 5.1 vs − 3.3). Preliminary evidence suggests that a supportive virtual community, accessed through a clinic-affiliated smartphone app, can help people living with HIV to address stigma.

## Resumen

El estigma tiene consecuencias negativas para la calidad de vida y los resultados de la atención del VIH. PositiveLinks es una intervención de salud móvil que incluye un tablero de mensajes de comunidad anónimo seguro (CMB por sus siglas en ingles). Investigamos la discusión sobre el estigma y los cambios en los puntajes de estigma. De los 77 participantes en nuestra prueba piloto, el 63% eran hombres, el 49% eran de raza negra y el 72% tenían ingresos por debajo del nivel federal de pobreza. El 21% de las publicaciones de CMB (394/1,834) contenían contenido relacionado con el estigma, incluidas publicaciones negativas (experimentando estigma) y positivas (superando el estigma) que abordaban el estigma intrapersonal e interpersonal. El estigma inicial más alto se correlacionó positivamente con el estrés y se correlacionó negativamente con la autoeficacia del cuidado del VIH. Los datos de 12 meses mostraron una tendencia hacia puntajes de estigma mejorados para quienes publicaban en el CMB versus quienes no publicaban (− 4.5 vs − 0.63) y para quienes publicaban contenido relacionado con el estigma versus otro contenido (− 5.1 vs − 3.3). La evidencia preliminar sugiere que una comunidad virtual de apoyo, a la que se accede a través de una aplicación de teléfono inteligente afiliada a la clínica, puede ayudar a las personas que viven con el VIH a enfrentar el estigma.

## Introduction

Despite recent advances in HIV care, stigma against persons living with HIV (PLWH) remains common and can have a negative impact on quality of life and overall health [1, 2]. In the United States, HIV-related stigma is particularly prevalent in rural areas and in the South [3–8]. HIV-related stigma can be complicated by other sources of stigma that PLWH also experience based on gender, sexual orientation, race or ethnicity, poverty, substance use, or mental illness [9–11]. The criminalization of HIV transmission further contributes to stigma and discrimination against PLWH [12, 13]. Stigma is associated with poor physical and mental health for PLWH [14–18], lower medication adherence [19–21], and lower linkage to and retention in HIV care [22–24]. The mechanisms of stigma’s effects are complex and include both intrapersonal and interpersonal components [25–29]. Internalized stigma can impair one’s sense of self-worth and motivation for self-care, while experiences of discrimination and anticipated negative interactions can limit opportunities for social connection and access to healthcare [25]. Stigma also plays a role in disclosure decisions and can exacerbate social isolation and lack of support [30, 31].

Prior interventions to reduce HIV-related stigma among PLWH have focused on improving knowledge and attitudes about HIV, social support, and positive coping skills [32–36]. Although short-term results have been encouraging, there is a lack of high quality data regarding changes in behavior, long-term follow-up, or health outcomes [37]. In recent years, web-based and mobile health tools have gained attention in addressing stigma and social support. Emerging evidence suggests that stigma related to mental illness can be mitigated using virtual support group strategies [38–42]. Through online connections, individuals can overcome social isolation and gain support from others facing similar challenges, while maintaining anonymity. For PLWH, virtual communities can provide social support and empowerment [43, 44]. One web-based intervention, www.hivstigma.com, has demonstrated increased awareness of stigma against PLWH and a reduction in stigmatizing attitudes and behaviors [45].

For PLWH, mobile health tools have the potential to address multiple barriers to optimal care. Text messaging interventions can improve antiretroviral medication adherence, clinic appointment attendance, and clinical measures of CD4 counts and viral loads [46–50]. Smartphone applications (apps) provide more functionality and security than text-based tools but most apps currently available for PLWH are not rigorously evidence-based and do not specifically address issues of stigma [51]. The PositiveLinks intervention was designed to address challenges for PLWH in achieving retention in care and favorable clinical outcomes, specifically targeting a vulnerable patient population in the rural south of the United States [52, 53]. Through an iterative, user-driven process, our team developed the PositiveLinks smartphone app which includes appointment reminders, daily queries of mood, stress, and medication adherence, tailored educational resources, access to the study team for individualized counseling and assistance, and the opportunity to interact with other users on a secure anonymous community message board (CMB).

The development of PositiveLinks was informed by our team’s prior experience with text-based mobile interventions for PLWH [46, 47] and formative work with our target users [54–56]. Our methods included interviews with clinic patients regarding needs and preferences for app features, population-based design customization, and preliminary testing with our users, consistent with the emerging literature on mobile app design for PLWH [56–60]. Our formative work and similar investigations in other populations of PLWH [61–63] have revealed a desire for social connection and interactive engagement as key features of mobile apps, in addition to monitoring functions, reliable information, and privacy protection.

The ultimate goal of the PositiveLinks intervention was to improve retention in care and clinical outcomes for PLWH. Stigma was targeted as a known modifiable mediator of retention in care [22–24]. The CMB feature was proposed to serve as a source of social support and community acceptance with the potential to influence participants’ perceptions of stigma. We made the CMB available to all participants through the app, but use of this feature was optional. We followed the formation of the PositiveLinks community prospectively to observe whether the CMB provided PLWH a safe space to address stigma. We anticipated that, by forming a positive community, participants could find acceptance and regain an internal sense of worth and a hopeful view of their future.

The objectives of this study were to understand the discussion of stigma within the virtual community of the CMB and to evaluate change in participants’ stigma levels after 12 months of follow-up. We hypothesized that stigma levels would decrease, particularly for PositiveLinks participants who posted on the community board.

## Methods

### Enrollment

Participants were recruited through provider referrals at the University of Virginia Ryan White HIV Clinic and from local AIDS service organizations and HIV testing sites. Inclusion criteria were HIV positive status and age over 18 years old. Participants were then excluded if they were unable to achieve a score of 40 on the Wide Range Achievement Test (WRAT-4) or pass a subsequent reading test corresponding to an approximately fourth grade reading level [64]. This testing was performed at enrollment. The app was designed to accommodate low literacy and only four prospective participants were excluded due to concern that insufficient literacy would limit use of the app. Participants were not required to own a phone to be eligible for enrollment.

Participants were either newly diagnosed with HIV (within 90 days of enrollment), returning after a lapse in care, or at risk of falling out of care (as determined by their HIV care provider). HIV care providers determined a lapse in care or risk of falling out of care based on their experiences, including history of missed appointments, challenges with adherence, and other psychosocial barriers that complicated care. During enrollment, participants completed written informed consent and a baseline questionnaire. Data collection included participant demographics, socio-economic status, and previously validated measures for perceived stress [65] and self-efficacy for starting care, staying in care, keeping appointments, and medication adherence [66]. Stigma was measured using the Berger Stigma Scale at baseline and at 12-month follow-up [67]. Clinical data were extracted from the electronic medical record to obtain CD4 counts and viral loads for participants. Viral suppression was categorized as less than 200 copies/mL, consistent with the HRSA quality measure definition [68]. Enrollment occurred on a rolling basis between September 2013 and May 2015. IRB approval from the University of Virginia was obtained for the study.

During enrollment, regardless of prior phone ownership, participants were given Samsung Galaxy 2 or Galaxy 3 smartphones with the PositiveLinks app installed and completed a tutorial on how to use the phone and app. PositiveLinks is a native Android app that was installed on the participants’ study phones. Participants new to smartphone use were provided additional training and assistance to ensure understanding of app usage as well as basic phone functions. Enrollment in the study included a talk, text, and data plan. The phones and app were encrypted, password protected, and had a remote locate and wipe functionality.

Participants could interact with anonymous usernames, created during enrollment, on the CMB through the app. Expectations for appropriate use of the CMB were reviewed at enrollment with emphasis on respectful dialogue with other users. The consent and enrollment process specifically addressed privacy risks. Participants were advised not to reveal personally identifying information on the CMB. Posts made by participants were viewable immediately on the CMB. The PositiveLinks study team monitored the CMB for misinformation or inflammatory comments and could reach out to participants privately if needed by phone or text. The team accessed the CMB through a web-based administrative portal and checked it daily, including weekends and holidays. Inappropriate posts or disclosures of personal information were deleted and the poster was notified and reminded of the importance of anonymity and respect. However, inappropriate posts were a rare occurrence and no participants were removed from the study for CMB misuse.

The study design was a prospective mixed methods study. First, qualitative analysis of the CMB content was performed. Second, participants were categorized based on their CMB use and quantitative analysis was conducted to assess pre-post changes in stigma scores for the entire group and by CMB-use subgroups. Details for each step are provided below.

### Qualitative Analysis of the Community Message Board

Messages were analyzed qualitatively using constant comparative analysis [69]. Each post could be assigned more than one code if several topics were expressed. Posts were classified as stigma-related if they referred to social consequences of HIV status. Coding was performed by 2 independent coders on 20% of the dataset to develop the codebook and establish inter-coder reliability. The codebook was refined until excellent reliability was achieved, with a kappa statistic of 0.86. The codebook was then applied to the entire dataset of posts so that the frequency of each topic category could be evaluated.

Validity of the coding system and analysis was assessed through discussion with the study team, composed of experts in infectious disease, clinical psychology, and public health sciences. Posts were divided into four major categories based on whether the content related to interactions with others (interpersonal) or thoughts of oneself (intrapersonal), as well as whether the stigma was being experienced (negative) or overcome (positive) by the poster. Subcategories were developed within these four groups. Conversation threads on the CMB were also examined in order to see the posts in context and to observe interactions among participants in response to stigma-related themes. Participants’ responses to stigma-related themes were categorized as positive, negative, or neutral. This approach was consistent with intra- and interpersonal conceptualizations of stigma and the experience of stigma within the context of community [25–29]. Analysis was performed using NVivo qualitative data analysis software (QSR International Pty Ltd. Version 11, Sept 2015).

### Quantitative Analysis of Stigma Scores

The primary outcome of interest was HIV-related stigma, measured at baseline and at 12 months by the Berger Stigma Scale [67]. This scale assesses stigma perceived by PLWH, including personalized stigma, negative self-image, disclosure, and public attitudes subscales. Participants respond to statements about stigma experience using a scale from 1 (strongly disagree) to 4 (strongly agree) with a total score calculating from the sum of 40 items. Possible scores range from 40 to 160 with higher scores indicating higher levels of stigma. The primary independent variable was posting behavior on the CMB, as determined by the qualitative analysis. Participants were categorized as non-posters, posters of stigma-related content, and posters of non-stigma-related content. Co-variates included age, gender, race, risk behavior, income, and education levels. Correlations between stigma levels and scores on stress or self-efficacy measures were also assessed.

A dependent t test was used to compare participants’ baseline stigma and 12-month stigma scores. One-way ANOVA tests (p-value from F-test) were used to compare changes in stigma between those who posted to the CMB and those who did not and to compare changes in stigma between those who posted on the CMB, but not related to stigma, and those who made stigma-related posts. Pearson correlation coefficient tests and one-way ANOVAs were used to determine if higher baseline stigma scores were associated with stress and self-efficacy or demographic factors. Associations between change in stigma and demographics was also assessed with one-way ANOVA tests. All significance was evaluated at an alpha level of 0.05. Analyses were performed using SAS 9.4 and GAUSS 16.0.

## Results

### Participant Characteristics

Table 1 shows participant characteristics. Among the 77 participants in the study, 63% were male; 49% identified as Black, 34% as White, and 8% as Hispanic. The majority of participants (58%) reported income below 50% of the Federal Poverty Level and 45% had a high school diploma or GED as their highest level of education. For HIV transmission risk factors, 48% reported heterosexual behavior, 40% identified as Men Who Have Sex with Men (MSM), 7% reported injection drug use, and 3% were transgender male to female. At baseline, 47% of participants had achieved viral suppression (viral load < 200).

**Table 1:** Participant characteristics

	Total (n = 77)
Gender, n (%)	
Male	49 (64)
Female	26 (34)
Transgender male to female	2 (3)
Race/Ethnicity, n (%)	
White non-hispanic	26 (34)
Black non-hispanic	38 (49)
Hispanic	6 (8)
Asian	1 (1)
Multiple races	5 (6)
Refused	1 (1)
Income compared to federal poverty level, n (%)	
0% ≤ FPL < 50%	45 (58)
50% ≤ FPL < 100%	11 (14)
100% ≤ FPL < 150%	12 (16)
150% ≤ FPL < 200%	5 (6)
200% ≤ FPL	4 (5)
Risk factor, n (%)	
Heterosexual	37 (48)
IV drug use (IDU)	3 (4)
IDU/MSM	2 (3)
Men who have sex with men (MSM)	31 (40)
Transgender	2 (3)
Don’t know/missing	2 (3)
Level of education, n (%)	
≤ 6 years	1 (1)
7–11 years	14 (18)
High school graduate	27 (35)
GED	8 (10)
Community college	2 (3)
Trade or technical school	4 (5)
Some college	15 (19)
College graduate	6 (8)
Enrollment characteristics, mean (SD)	
Months from HIV diagnosis to enrollment	60 (76)
Age in years at enrollment	36 (12)
Clinical characteristics, mean (SD)	
Baseline CD4+	522 (373)
Baseline VL	23682 (60820)
Baseline log10(1 + VL)	2.46 (1.79)
Baseline Appointment Adherence	85 (23)
Baseline stigma scores, mean (SD)	102.94 (18.26)

### Stigma-Related Content on the Community Message Board

**Fig. 1:**
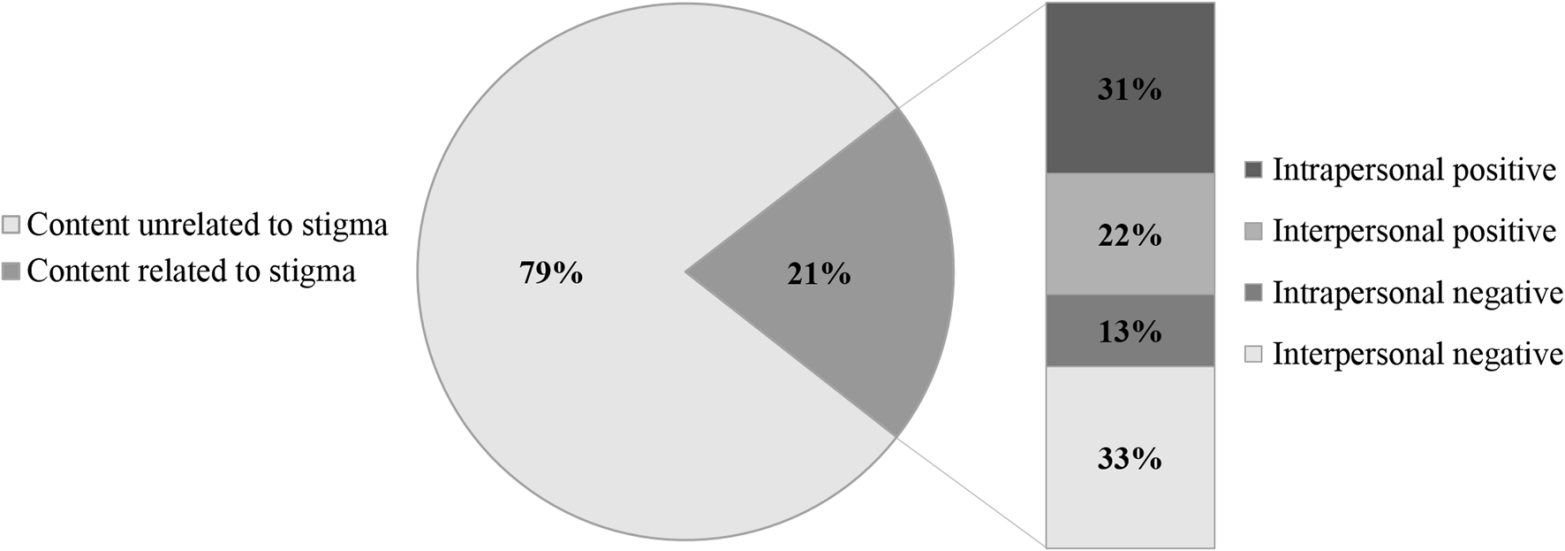
Community message board coding categories and frequencies

Over 30 months, 2300 posts were made on the CMB. Posts from the PositiveLinks study team, blank posts, and duplicate posts were excluded, leaving 1834 posts for analysis. Of these 1834 posts, 21% (394 posts) contained content related to stigma. Figure 1 shows the frequency of each major stigma category: intrapersonal positive content (31%), interpersonal positive content (22%), intrapersonal negative content (13%), and interpersonal negative content (33%). These categories were further analyzed and Table 2 shows each sub-category with the frequency of occurrence. For quotations from posts, participants’ non-standard spelling and grammar were retained in order to present their experiences in their own words.

**Table 2:** Stigma themes and frequency of occurrence

Stigma themes	% stigma-related posts (n)
Experiences of stigma (negative)	
Intrapersonal	
Negative framing of HIV + status	7% (26)
Lack of self-worth	6% (24)
Interpersonal	
Feelings of loneliness and isolation	9% (34)
Disrupted relationships	8% (30)
Negative past experience with disclosure	6% (24)
Negative anticipated experience with disclosure	6% (22)
Fear of transmission	3% (10)
Negative consequences of failing to disclose	2% (6)
Overcoming stigma (positive)	
Intrapersonal	
Positive reframing of HIV + status	18% (72)
Affirming self-worth	12% (46)
Interpersonal	
Finding “true” friendship/love/family	10% (38)
Positive past experience with disclosure	9% (35)
Positive anticipated experience with disclosure	3% (11)

#### Experiencing Stigma

Intrapersonal negative content described participants’ internalized experiences of stigma, which included posts referring to lack of self-worth (7% of stigma-related posts) and negative framing of HIV status (6%). In the lack of self-worth category, participants described feelings of worthlessness and feeling undeserving of love due to HIV status. For example, one participant stated: “I just found out I’m h I v positive 5 days ago and the women I got it from treat me like s*it and I’m in va with no fam and she tell me i’ll never get nobody eles bc I have h I v…this is the first time I felt bad.” In posts with negative framing, participants suggested that HIV status was a result of past actions or a negative aspect of one’s life. These could include feelings of regret, punishment, or feelings of being doomed or cursed. One example was, “I died when I found out, I’m still dead. I am here to be punished.”

Interpersonal negative content described participants’ experiences of stigma in their relationships with others. Sub-categories included feelings of loneliness and isolation (9%), disrupted relationships (8%), negative past experiences with disclosure (6%), negative anticipated experiences with disclosure (6%), fear of transmission (3%), and negative consequences of failing to disclose (2%). The most common among these, feelings of loneliness and isolation, described both physical separation from others and the psychological isolation of living with HIV. “I’m wondering if anyone here has suffered a significant loss that has turned life upside down. Not only am I dealing with being HIV positive but my partner died in May of this year and I’m still having a hard time grasping it. Without my partners positivity and love I’m feeling lonely and worthless about being HIV positive. How did others deal with this? (I feel like my road is at an end.)” Disrupted relationships were also frequently described; these were relationships disrupted as a result of HIV status and could be problems with friends or with a partner. “Okay so I’m currently stay in with my mom and its not working out……her and I have never really gotten along and ever since she found out my status its only gotten worse….” Participants’ posts about disclosure included past negative experiences and anticipated negative experiences. For example: “I feel like my supposed to be friend playing with my feelings n I opened up and told him that I have hiv n he said he wont tell anybody but he told two of his friends and I have not stayed home in a week now” and “mayb I havent n dont tell n e one bcuz I AM afraid of rejection or being treated differently”. Fear of transmission included posts describing fear of transmitting HIV through intercourse, IV drug use, or blood exposure. These fears could relate to either the poster’s own actions or those of another. “My fiance is still negative n he refuses to cover which makes it so difficult for me to relax n enjoy that moment.. iv done almost everything to push him away becuase im afraid of infecting him…” Negative consequences of failing to disclose were also seen on the CMB and described loss of trust as well as ethical or legal consequences. For example, when a participant mentioned their husband, who was HIV positive, was engaging in extra-marital sexual activity and was failing to disclose his status, another participant asked, “What ur husband is doin is AGAINST THE LAW does the medical community know of this?”

#### Overcoming Stigma

Participants posted positive content that described overcoming or rejecting stigma, both in their self-image (intrapersonal) and their relationships with others (interpersonal). Of intrapersonal positive content, posts described positive reframing of HIV status (18%) and the concept of affirming self-worth (12%). Positive reframing of HIV status described posts where users reframed living with HIV in a good way. These posts described a conscious decision to not let HIV negatively affect their lives, but to instead embrace it as a part of their identity or to allow it to make positive changes in their life. “I strive to look at things in my life for the better and show my three children that any thing is possible if you have faith. Me being. Hiv positive hasn’t changed me at all.” Posts affirming self-worth expressed the idea that one still has value despite HIV status and to the belief that one deserves love and respect from others, God, and/or themselves. For example, one user wrote, “Like I always said there is hope I dont kno y ppl give up when they get H.I.V. I don’t kno the word quit I just can’t bc I love me to much and I don’t just say I believe in GOD I really do and so should you”.

Of interpersonal positive content, posts included that their HIV status had allowed them to identify deeper relationships (10%), as well as posts that described positive past experience with disclosure (9%) or anticipated positive future experiences with disclosure (3%). Posts that described finding deeper relationships suggested that their HIV status allowed them to find “true” friendships, love, or family, and included relationships within the PositiveLinks community and/or the larger HIV + community. One example of support from the PositiveLinks family was, “Over time, my strength prevailed! I’m still growing with the new knowledge of knowing but with the support of all The Positive link family have made me realize I still have a future. I truly believe we can all prevail together our new positive family member.” Posts dealing with disclosure described both positive past experiences and positive anticipated experiences. For example: “I wonder how everyone here felt when they had to tell their ex’s of the one they were or are with when the told them. I had to do that today and I never been this nervous in a long time. When I told the I coulsd of died right there and then. Weird thing is it seemed to drop the tenchen between us all most instantly. Very strange day.” Positive future experiences could come from the initial poster anticipating a disclosure conversation going well or from another participant encouraging the initial poster to disclose his/her status to someone else, such as: “if u want to talk to your friend about your HIV status it’s ok if he is indeed your friend he should understand and it shouldn’t change your friendship and it will make u feel better to talk to someone close to you. I had to do the same it was hard but once I got it out I felt better and he may have questions about just be honest and let him know the facts of being HIV positive.”

#### Interactions Among Participants in Stigma-Related Threads

Posts containing stigma-related content and their responses were also classified in order to characterize how users in the virtual community interacted with each other in discussing stigma themes. Posts and their responses were grouped by if they were positive, neutral, or negative, and again, posts could be assigned more than one code if several different themes were expressed. Table 3 shows each category of thread interactions and its frequency of occurrence.

**Table 3:** Interactions among participants in stigma-related threads

Thread Interactions	% stigma-related posts (n)
Positive	
Companionship	51% (204)
Positive thinking/purpose	30% (118)
Blessings/affirming God’s love	13% (53)
Will to overcome	13% (51)
Neutral	
Sharing one’s story	24% (95)
Instructional advice	11% (45)
Asking a question	6% (23)
Sharing need to talk	2% (6)
Negative	
Negativity	14% (54)

Posts that were identified as positive thread interactions included posts expressing companionship (51% of stigma-related posts), positive thinking/purpose (30%), blessings/affirming God’s love (13%), and the will to overcome (13%). Companionship posts offered solidarity, friendship, and support to another member of the CMB, either individually or from the entire PositiveLinks community, and occurred with the greatest frequency of any stigma-content theme. “I know how u feel but I/the positive family love u more then u know….. We are here for u n will always have ur back no matter what. So remember one love.” Positive thinking/purpose also occurred with a high frequency; these posts shared strong positive emotions or emphasized the importance of positive thinking in relation to one’s HIV status. These could include concepts of purpose or destiny, either innate or given by God, or the suggestion that a difficult situation would improve with time: “Along with the positive results from the doctor, we have to keep a positive attitude…..And our faith. Because THAT’S invaluable.” Blessings/affirming God’s love were often messages of support to another CMB member, such as “So sorry u r feeling bad about life an everything else I have been there myself so I know what u mean. Just have to take one day at a time an keep god by your side. He will help u to be strong. It will get better for u. It just takes time.but I feel for Ya.” Will to overcome occurred at the same frequency as blessings and was defined as posts encouraging another member of the CMB to keep fighting against the struggles they are facing, to be strong, and/or to not give up: “Just chin up and ride this storm out. Together we stand and remain strong we all can get past this one day at a time.”

Neutral post types included sharing one’s story (24%), instructional advice (11%), asking a question (6%), and sharing the need to talk (2%). Sharing one’s story often occurred as a way to start discussion on stigma or to connect with another member of the board who had a similar experience with stigma. One example of this was “Hello. I just Jumped into them. Trying to deal with this alone was really just too stressful. Finally told three people I trust. It does get easier. Even though it has barely been a month dealing with this.” Instructional advice was defined as one participant offering specific advice to another member of the CMB about actions to take in dealing with stigma (i.e., specific coping strategies, safe sex practices, and ways to deal with disrupted relationships.) For example, “It’s hard to do (disclosure) but u just have to anyway. Also you can have ur case worker make an appointment with u and the person you are dating n tell him or her to make things easier for you.” Asking a question was also observed; “How do you find the courage to tell someone that you are datIng that you have Hiv?” Participants also shared the need to talk about stigma-related issues, “Im so mad and not sure what to do Im really tired of feeling like im beening use and played.I hate when ppeople play with ur emotions n feelings. Need someone to talk to.”

Negative thread interactions were grouped into one category (14%), which contained posts expressing strong negative emotions on the CMB or posts containing a negative reaction to another member’s post(s). One such example was “Sure sucks when u get this diease an that person doesn’t even tell u they have it.. a sucky situation like I said.im a very angry person right now.”

### Results of Stigma Scores

Of the 77 participants, 53 completed both the baseline and 12-month stigma questionnaires. In this sample, participants’ stigma scores showed a mean of 102.94 (SD 18.26) at baseline and 98.73 (SD 15.08) at 12 months. There was a trend toward reduced stigma with a mean change of − 3.9 (95% CI − 8.1, 0.2) but it was not statistically significant (p = 0.060, dependent t test).

Participants were grouped into non-posters (n = 20), posters who never posted stigma-related content (n = 15), and posters who did post stigma-related content (n = 42). Non-posters had a mean change in stigma scores of − 0.63 (SD 9.2) as compared to − 4.5 (SD 15.7) for posters. Among those who posted, posters of content unrelated to stigma had a mean change in stigma scores of − 3.3 (SD 12.7) as compared to − 5.1 (SD 17.2) for posters of stigma-related content. There was a trend toward more improvement in stigma scores with posting versus not posting and with posting about stigma versus other content, though these differences were not statistically significant (p = 0.500 and p = 0.722 respectively, one-way ANOVA F-test).

Baseline stigma scores showed moderate positive correlation with perceived stress score (PSS) (0.51, p < 0.001). There were moderate negative correlations between stigma and self-efficacy to start HIV care at − 0.46 (p < 0.001), and between stigma and self-efficacy to stay in HIV care at − 0.39 (p = 0.001). As stigma increased, self-efficacy to start and stay in care decreased. There was a small negative correlation between stigma and self-efficacy to attend appointments at − 0.26 (p = 0.025), meaning that as stigma increased, self-efficacy to attend appointments decreased. Stigma scores were not significantly correlated with self-efficacy for medication adherence. These results can be seen in Table 4.

**Table 4:** Correlation of baseline stigma with other baseline measures

Baseline measure	Correlation	95% CI	p value*
Perceived stress	0.51	(0.32, 0.66)	< 0.001
Self-efficacy to start HIV care	− 0.46	(− 0.62, − 0.25)	< 0.001
Self-efficacy to stay in HIV care	− 0.39	(− 0.57, − 0.18)	0.001
Self-efficacy to attend HIV appointments	− 0.26	(− 0.46, − 0.03)	0.025
Self-efficacy to take HIV medications	− 0.11	(− 0.33, 0.12)	0.330

*p values from Pearson correlation analysis

Finally, we found that stigma scores at baseline had no significant associations with any of the demographic characteristics assessed: age, race, gender, education, income, or transmission risk. However, a change in stigma between baseline and 12 months was significantly associated with gender (p = 0.047, one-way ANOVA F-test). Men had a significantly greater decrease in stigma (− 7.1, SD 14.9) than women (1.3, SD 13.8).

Assessment of possible impact of missing data was also performed. Twenty participants were missing Berger Stigma Scores at 12 months, of which 8 were non-posters, 12 posted stigma-related content, and none posted only non-stigma-related content. Baseline stigma score was a significant predictor of 12-month stigma scores (p < 0.001, coefficient = 0.505, SE = 0.10). Subsequently, imputed values were substituted for the missing 12-month stigma scores. Using 50 multiply-imputed datasets, with baseline stigma as a covariate, there was still no significant change between baseline and 12-month stigma scores across groups, p = 0.96 (one-way ANOVA F-test).

## Discussion and Conclusion

### Discussion

Stigma remains a pervasive and challenging problem for PLWH with negative consequences for wellbeing and health. In this study, PositiveLinks participants expressed internalized stigma with feelings of lacking self-worth and seeing their HIV status as a curse or a punishment. They also experienced stigma in their relationships, complicating disclosure decisions and exacerbating a sense of isolation. Participant responses indicated stigma scores comparable to similar populations of PLWH [67, 70]. Stigma scores were correlated with worse stress and self-efficacy for HIV care. However, several encouraging findings were also observed. Members of the PositiveLinks community expressed ways of overcoming stigma through affirming self-worth and reframing HIV status in a hopeful light. They described opportunities to find true friends and family and encouraged disclosure as a way to deepen relationships and seek support. The majority of interactions among participants in stigma-related threads were positive, with participants offering companionship and understanding to those community members who were struggling with stigma-related challenges.

There was a trend toward improved stigma scores at 12 months, with more improvement seen among those who posted on the message board, and particularly those who posted stigma-related content. Though this was a pilot study with small numbers, this finding suggests a positive impact for a virtual support group as part of a feasible and acceptable intervention to address HIV-related stigma. This study builds on prior work indicating that virtual support groups can improve stigma in mental illness through building the self-confidence of posters and creating a community of acceptance and shared identity [38–42].

While most online mental health support groups have been predominantly female [38, 39], our study population was primarily male. Interestingly, changes in stigma scores were more favorable for males. It is not clear why this gender difference in stigma outcomes occurred. In a majority male group, male participants may have felt more comfortable than females in posting and therefore had more opportunities to derive benefit from the community’s social support. However, several instances were noted in the CMB discussions in which participants mistakenly assumed each other’s genders and respectfully corrected each other. Their user names were often ambiguous or gender-neutral. It is also possible that the community was particularly welcoming to MSM from a non-urban area, making the CMB more valuable to them in overcoming stigma.

Stigma may also play a role in the willingness of PLWH to participate in private, secure mobile health interventions. Age, HIV stigma, and social isolation have been negatively associated with smartphone use in research studies [71]. Patients most at risk for stigma may also be most difficult to reach with mobile health interventions, which should be taken into consideration when seeking to address stigma through app-based communities. Of note, we have no evidence to suggest that PLWH with more stigma chose not to enroll in PositiveLinks.

The PositiveLinks intervention is unique in targeting a vulnerable population of PLWH in the rural southern United States, incorporating low literacy into the design, and embedding the virtual community within a smartphone app. In addition to the CMB, the PositiveLinks app provides medication and mental health tracking, reminders, educational resources, and messaging with the study team. Although the CMB was the app feature most directly designed to address stigma, it is possible that app engagement in general could provide benefit. In this pilot, we could not assess the influence of different app features. The intervention is also affiliated with the participants’ source of care at the Ryan White HIV clinic, providing a connection not only to peers on the CMB but also to HIV care providers. Stigma acts at multiple levels, including individual, interpersonal, institutional, and societal impact. Interventions to address stigma may be more effective if multiple levels are addressed [37]. PositiveLinks may act at the individual level (through fostering self-care and a positive self-image) and the interpersonal level (through enabling a supportive virtual community for PLWH), and to some extent at the institutional level (through incorporation into the HIV clinic). Further work at the institutional level is planned as PositiveLinks expands to usual care at the clinic to reduce barriers to care for PLWH.

The CMB was a mostly passive intervention on the study team’s part, providing an opportunity for participants to create a virtual community. Participants’ active use of the CMB was required to achieve an effect. Although we expected from our formative work that our participants would value this opportunity and use it as intended, there was no guarantee that this would occur. The community was created by PLWH for themselves and their peers. The PositiveLinks team monitored the CMB and could intervene if needed, but the community was primarily self-regulating. Users modeled affirming and supportive behavior to each other.

Other virtual support groups for PLWH have shown mixed results with some improvements in psychological health among participants [43] but also with potential for disempowerment [72]. Negative features of publicly available groups, including misinformation and inappropriate interactions, may be mitigated in a closed and monitored group like PositiveLinks. Non-virtual support groups have demonstrated benefit for PLWH in addressing stigma [36] but are difficult to implement in nonurban settings with poor transportation and low socioeconomic status. Our CMB provides a means to achieve the benefits of social support despite barriers to in-person groups. A reduction in stigma from the PositiveLinks intervention is likely a secondary effect of social interaction in the virtual community. The CMB can fill a need for affirmation and acceptance for PLWH who face rejection from other social groups. Stigma is important to address because of its impact on mental health and quality of life and also for long-term implications as a mediator of retention in care and clinical outcomes.

The study has several limitations to consider. First, this was a pilot study with a small sample size that was likely underpowered to detect differences in stigma scores, especially between subgroups. Pilot studies can provide preliminary information on feasibility and acceptability but should not be used to draw conclusions for hypothesis testing or effectiveness [73, 74]. Second, the study was a single-arm prospective design without a control group. Therefore, conclusions about causation cannot be drawn and it is not possible to determine the differential effects of the app features. Third, PositiveLinks is affiliated with a specific care setting, which may limit generalizability. Fourth, enrollment criteria relied on provider referral which could be subjective and based on clinical judgement about patients’ risk for poor retention in care. Finally, a focus on HIV-related stigma does not fully address the multiple layers of stigma that many PLWH experience. It is important to recognize that PLWH may have several co-occurring stigmatized identities beyond their HIV status, such as gender, race, drug use, poverty, mental illness, and sexual minority status. Systemic discrimination based on these stigmatized identities persists, despite interventions to address more proximal intra- or interpersonal levels.

### Conclusion

The PositiveLinks mobile intervention allows participants from vulnerable populations to express both positive and negative concepts of stigma and to find support within a virtual community. Next steps will include expansion of PositiveLinks to the entire Ryan White clinic at the University of Virginia and to other sites. More robust statistical analysis will be possible in a larger population and with additional longitudinal follow-up. In addition, examination of community message boards with new members and at other sites will demonstrate the degree to which our pilot CMB is replicable and how best to adapt the PositiveLinks intervention to the needs of other populations. Larger studies with randomization will be necessary to establish the efficacy of PositiveLinks and investigate differential effects of the intervention components such as the CMB.

Virtual support groups have the potential to assist PLWH in rejecting stigma and overcoming social isolation. Through fostering a positive self-image and supportive relationships, these communities can help to mitigate stigma and ultimately improve quality of life and health outcomes.
